# The association of body mass index and body composition with plasma amyloid beta levels

**DOI:** 10.1093/braincomms/fcad263

**Published:** 2023-10-09

**Authors:** Marco Hermesdorf, Hermann Esselmann, Barbara Morgado, Anke Jahn-Brodmann, Marisol Herrera-Rivero, Jens Wiltfang, Klaus Berger

**Affiliations:** Institute of Epidemiology and Social Medicine, University of Münster, Münster 48149, Germany; Department of Psychiatry, University Medical Center Göttingen, Goettingen 37075, Germany; Department of Psychiatry, University Medical Center Göttingen, Goettingen 37075, Germany; Department of Psychiatry, University Medical Center Göttingen, Goettingen 37075, Germany; Department of Genetic Epidemiology, Institute of Human Genetics, University of Münster, Münster 48149, Germany; Department of Psychiatry, University of Münster, Münster 48149, Germany; Department of Psychiatry, University Medical Center Göttingen, Goettingen 37075, Germany; German Center for Neurodegenerative Diseases (DZNE), Goettingen 37075, Germany; Neurosciences and Signaling Group, Institute of Biomedicine (iBiMED), Department of Medical Sciences, University of Aveiro, Aveiro 3810-29992, Portugal; Institute of Epidemiology and Social Medicine, University of Münster, Münster 48149, Germany

**Keywords:** amyloid beta, plasma, body mass index, body fat, bioelectrical impedance analysis

## Abstract

Blood-based analysis of amyloid-β is increasingly applied to incrementally establish diagnostic tests for Alzheimer’s disease. To this aim, it is necessary to determine factors that can alter blood-based concentrations of amyloid-β. We cross-sectionally analysed amyloid-β-40 and amyloid-β-42 concentrations and the 40/42 ratio in 440 community-dwelling adults and associations with body mass index, waist-to-height ratio and body composition assessed using bioelectrical impedance analysis. Body mass index and waist-to-height ratio were inversely associated with plasma amyloid-β-42 concentrations. Body fat mass, but not body cell mass and extracellular mass, was inversely associated with amyloid-β-42 levels. The results indicate that plasma concentrations of amyloid-β-42 are lower in those with increased body mass index and body fat, and associations with amyloid-β-40 did not reach significance after controlling for multiple testing. The findings support the use of body mass index as an easy-to-measure factor that should be accounted for in diagnostic models for plasma amyloid-β.

## Teaser

Blood-based tests for amyloid-β are developed to enable a minimally invasive screening for Alzheimer’s dementia in the general population. It is important to identify factors that affect plasma amyloid-β levels. The current results revealed inverse associations between plasma amyloid-β, body mass index, waist-to-height ratio and body fat mass.

## Graphical Abstract

**Graphical Abstract:**
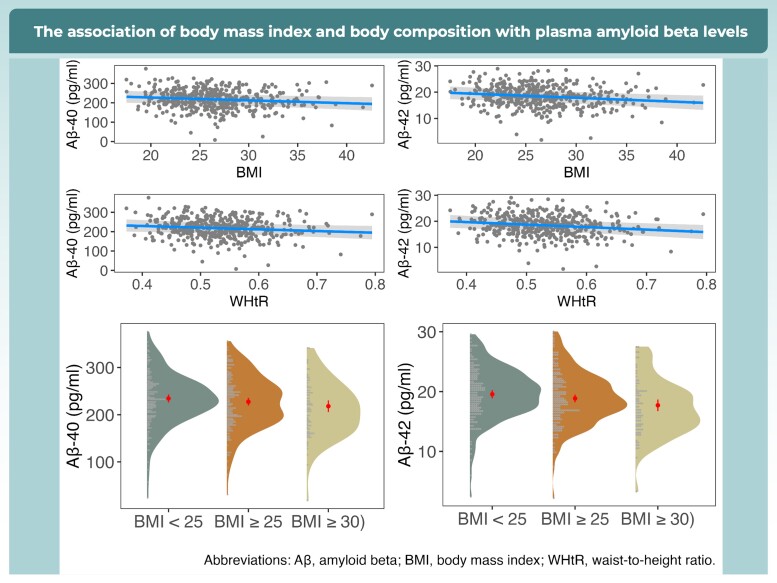

## Introduction

Alzheimer’s disease is a neurodegenerative disorder that is preceded by a long prodromal stage characterized by an initially subtle but progressive decline in cognitive abilities.^1^ Accumulating amyloid-β (Aβ) within the brain during disease progression is a hallmark of Alzheimer’s disease, although it is debated whether accumulating deposition of Aβ plaques has a causal effect on disease progression or is just a mere consequence of Alzheimer’s disease.^2^ Nevertheless, the accumulation of Aβ, particularly the isoforms Aβ-40 and Aβ-42, offers the chance to predict the conversion to Alzheimer’s disease and to monitor disease progression in those already affected by the disease.

Aβ accumulation is often quantified in the CSF^3^ and can also be assessed using PET.^4,5^ Recent advances enable the assessment of Aβ levels in serum or plasma using ultrasensitive technologies, which are less invasive compared to a PET scan or a lumbar puncture. This facilitates the analysis of Aβ levels particularly in large samples of the general population as a potential screening for the future emergence of neurodegenerative disorders.^6^ However, for this purpose, it is necessary to elucidate factors that potentially confound the association between blood-based biomarkers of neurodegeneration and disease or functional status. It has recently been shown that concentrations of serum neurofilament light, a marker of acute neuroaxonal damage measured using single molecular array technology, are inversely related to body mass index (BMI) and total blood volume.^7^ A detailed examination of body composition using bioelectrical impedance analysis (BIA) revealed that serum neurofilament light was inversely associated with body fat mass, body cell mass and total body water.^8^ This suggests that concentrations of proteins of neuronal origin assessed in the blood using ultrasensitive assays can be affected by confounding due to body composition, either because of individual differences in blood volume or specific tissue types absorbing the proteins of interest. Such confounding effects are particularly relevant in case of neurodegenerative diseases, e.g. in case of Alzheimer’s disease where those affected by the condition might lose weight in a prodromal disease stage before they eventually receive the diagnosis.^9^

We used data from the general population cohort of the BiDirect Study to analyse associations between plasma Aβ concentrations and anthropometric indices of obesity operationalized as BMI and waist-to-height ratio (WHtR). It was hypothesized that concentrations for both Aβ-40 and Aβ-42 are lower in obese participants. We additionally analysed body composition in relation to plasma Aβ concentrations and hypothesized that elevated body cell mass, body fat mass, body water and blood volume were paralleled by lower Aβ-40 and Aβ-42 concentrations. Given these hypotheses, no significant association with the Aβ ratio was expected.

## Materials and methods

### Participants

The general population cohort from the longitudinal BiDirect Study (conducted between 2010 and 2020) comprised of initially 911 individuals aged between 35 and 65 years who were randomly sampled from the local population register of the city of Münster (Germany) and invited to participate in a comprehensive examination programme. For the present analyses, we included community-dwelling adults from this cohort who participated in the first follow-up examination (*n* = 800) when the BIA examination was conducted. Participants who did not undergo BIA (*n* = 39), those without available Aβ levels (*n* = 283) or missing data on apolipoprotein E (APOE) genotypes (*n* = 38) were excluded. The final sample consisted of 440 participants. All participants gave written informed consent at each study visit; the BiDirect Study was conducted in compliance with the standards specified in the Declaration of Helsinki and approved by the ethics committee of the Medical Faculty of the University of Münster.

### Anthropometric indices, BIA and blood volume

Body weight, height and waist circumference of all participants were assessed in the course of the standardized BiDirect examination programme and used to derive BMI $\left(\frac{\mathrm{weight}}{{\mathrm{height}}^{2}}\right)$ and WHtR $\left(\frac{\mathrm{waist}\mathrm{circumference}}{\mathrm{height}}\right)$. A BIA 2000-S device (Data Input GmbH, Pöcking, Germany) was used to estimate body composition with regard to body cell mass, body fat mass, extracellular mass and total body water. Total blood volume was calculated according to the Lemmens–Bernstein–Brodsky^10^ equation ($\mathrm{weight}\times 70/\surd \left(\frac{\mathrm{BMI}}{22}\right)$).

### Plasma Aβ

Blood was drawn from non-fasting participants, sampled in EDTA tubes, centrifuged and stored frozen at −80°C until use. For further analysis, samples were thawed at room temperature, mixed and centrifuged for 10 min at 4350 × *g* in a swingout rotor to remove insoluble material. Plasma Aβ levels were assessed using a semi-automated IP immunoassay as previous described.^11^ In brief, Aβ peptides were immunoprecipitated using the CyBio FeliX liquid handling instrument (Roboscreen, Leipzig, Germany). A total of 200 µL plasma was mixed with 200 µL H_2_O, 100 µL of 5 × IP buffer concentrate (250 mM HEPES/NaOH, pH 7.4, 750 mM NaCl, 2.5% Igepal CA630, 1.25% sodium deoxycholate, 0.25% SDS and Complete Mini Protease inhibitor cocktail) and 25 µL of M-280 magnetic beads (Invitrogen/Thermo Fisher Scientific Waltham, MA, USA) coupled with 1E8 monoclonal anti-Aβ antibody (nanoTools, Teningen, Germany). Incubation was performed overnight at 4°C with continuous agitation at 1000 rpm on an Eppendorf ThermoMixer C (Eppendorf, Hamburg, Germany). Magnetic beads were washed 3× for 5 min with 1 mL of PBS/0.1% BSA and 1× for 3 min with 1 mL of 10 mM Tris/HCl, pH 7.5. Aβ peptides were eluted in 20 µL of PBS containing 0.05% Tween-20 by heating 5 min at 99°C and 1000 rpm. Then the eluate was diluted with 60 µL Diluent 35 [Meso Scale Discovery (MSD)]. The eluates were divided into two aliquots and stored at −80°C until the measurements on Aβ multiplex immunoassays.

Plasma levels of Aβ-40 and Aβ-42 were assessed using the commercially available MSD Aβ panel 1 (6E10) V-PLEX multiplex immunoassay kit (MSD, Rockville, MD, USA). After blocking step, 15 µL of each diluted IP eluate was incubated with 15 µL of the 6E10-sulfotag detection antibody solution for 2 h at room temperature. Plate was read on a MSD QuickPlex SQ 120 reader (MSD) following the manufacturer’s instructions. All samples were measured with two technical replicates on the same assay plate and handled by a standardized procedure according to the guidelines of the German Center for Neurodegenerative Diseases. Estimated Aβ levels were above the lower level of quantification. Aβ recovery from a control sample with spiked Aβ-40 and Aβ-42 was 88 and 85%, respectively, following elution. Within-box coefficients of variation were 5.1% for Aβ-40 and 4.7% for Aβ-42. Between-box coefficients of variation were 6.7% for Aβ-40 and 7% for Aβ-42.

### Apolipoprotein E

The APOE alleles were determined according to the genotypes for the rs429358 and rs7412 variants extracted from the BiDirect genome-wide genotype data set. Briefly, genome-wide genotyping was performed on DNA isolated from EDTA blood using the Infinium PsychArray BeadChip v1 (Illumina). After quality control, genotype imputation was performed using the 1000 Genomes Project phase 3 reference panel. Here, rs7412 was genotyped while rs429358 was imputed with INFO > 0.8. The APOE allele was then determined using the rs429358/rs7412 haplotype as follows: ɛ2 = T/T, ɛ3 = T/C and ɛ4 = C/C.

### Statistical analysis

Associations with plasma Aβ-40 and Aβ-42 levels as well as the Aβ-40/Aβ-42 ratio as the dependent variables were analysed using separate analyses of covariance (ANCOVAs) adjusted for age, sex, batch and APOE genotype (carriers of at least one copy of the ε4 allele). Obesity indices (BMI and WHtR) served as independent variables, respectively. Additionally, BMI as a categorical independent variable was analysed separately with respect to Aβ levels. BMI weight status with established cut-offs has emerged as a useful categorization^12^ regarding clinical outcomes. Body composition was evaluated according to the three compartment model^13,14^ with fat mass, cell mass and extracellular mass as independent variables. Associations with body water and blood volume as predictor variables were separately analysed. Two-tailed *P*-values below 0.05 were considered significant and controlled for multiple testing using false discovery rate (FDR).^15^ The statistical analysis was conducted using R version 4.2.2.

## Results

**Figure 1:**
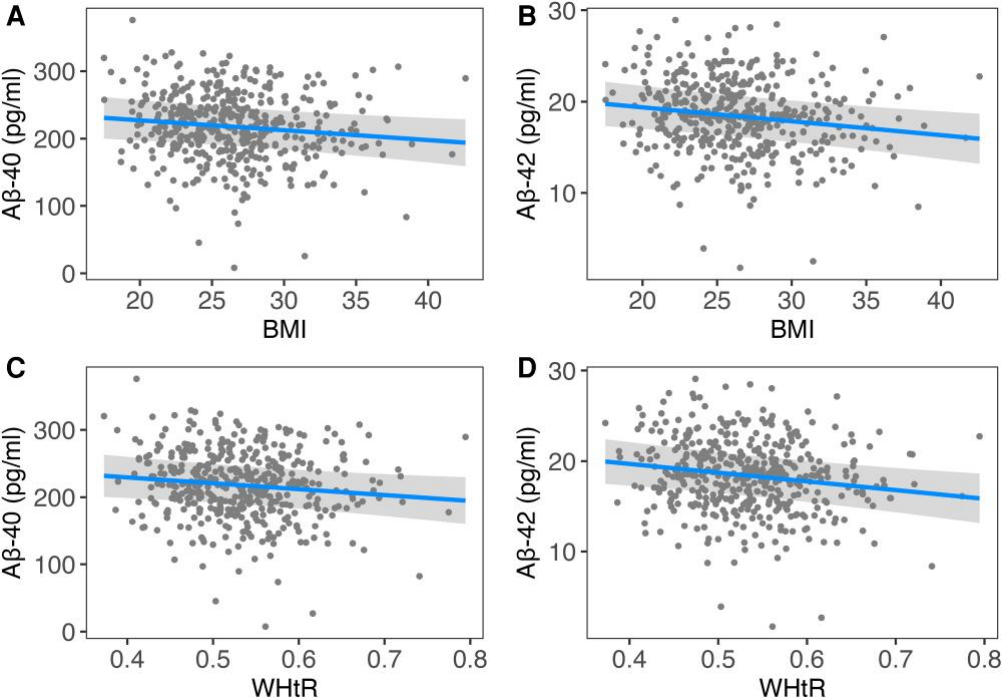
**Plasma Aβ concentrations and BMI.** Partial residual plots showing the adjusted associations of plasma Aβ-40 and Aβ-42 concentrations with the BMI (**A**, **B**) and the WHtR (**C**, **D**). Data points represent individual participants (*n* = 440), and the results were derived from ANCOVAs. Greyish shaded areas indicate the 95% confidence intervals. (**A**) *F* = 5.21, *P* = 0.023, *P*_corr(FDR)_ = 0.099. (**B**) *F* = 9.04, *P* = 0.003, *P*_corr(FDR)_ = 0.018. (**C**) *F* = 4.81, *P* = 0.029, *P*_corr(FDR)_ = 0.105. (**D**) *F* = 9.49, *P* = 0.002, *P*_corr(FDR)_ = 0.018). ANCOVAs, analyses of covariance; Aβ, amyloid-β; BMI, body mass index; WHtR, waist-to-height ratio.

**Figure 2:**
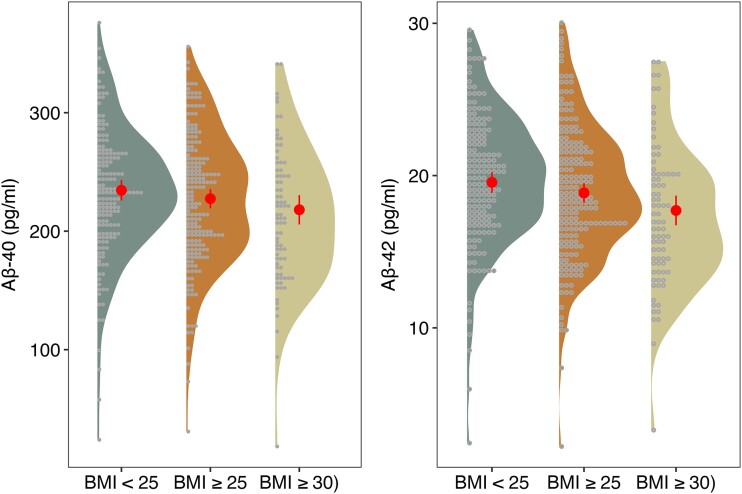
**Plasma Aβ concentrations and BMI categories.** Raincloud plots showing the distributions for plasma Aβ concentrations for different levels of obesity and estimated marginal means with 95% confidence intervals in red. Data points represent individual participants (*n* = 440), and the results were derived from ANCOVAs. Left panel: *F* = 2.44, *P* = 0.088, *P*_corr(FDR)_ = 0.191; right panel: *F* = 4.92, *P* = 0.008, *P*_corr(FDR)_ = 0.04. ANCOVAs, analyses of covariance; Aβ, amyloid-β; BMI, body mass index.

Demographic characteristics of the sample are shown in Table 1. The results revealed that elevated BMI was significantly associated with lower Aβ-42 but not Aβ-40 concentrations or Aβ ratio after accounting for multiple testing (Fig. 1 and Table 2). The same holds true for the WHtR, which was exclusively related to lower Aβ-42 levels. The categorical analyses of BMI yielded no significant association with Aβ-40 levels [*P* = 0.088, *P*_corr(FDR)_ = 0.191]. However, BMI groups were significantly related to Aβ-42 levels [*P* = 0.008, *P*_corr(FDR)_ = 0.04] with a significant contrast between participants with a BMI above 35 [*β* = −1.85, 95% confidence interval = −3 to −0.69, *P* = 0.002, *P*_corr(FDR)_= = 0.018] versus those with a BMI below 30 (Fig. 2). The analyses of BIA data revealed a significant inverse association between body fat mass and Aβ-42 but not Aβ-40 levels and the Aβ ratio (Table 3). Estimates for body cell mass and extracellular mass were not significant. The separate analyses of total blood volume and total body water did not show any significant associations with Aβ levels and ratio (all: *P* > 0.05).

**Table 1:** Sample characteristics

	Participants
	*n* = 440
Age (years): mean (SD)	56.38 (7.73)
Women: *n* (%)	215 (49%)
APOE-4 carriers: *n* (%)	113 (26%)
Aβ-40 (pg/mL): mean (SD)	226.11 (55.69)
Aβ-42 (pg/mL): mean (SD)	18.94 (4.45)
Aβ ratio (40/42): mean (SD)	11.95 (1.30)
Body mass index: mean (SD)	26.43 (4.15)
Waist-to-height ratio: mean (SD)	0.53 (0.07)
Body fat mass (kg): mean (SD)	24.18 (8.42)
Body cell mass (kg): mean (SD)	28.19 (6.35)
Extracellular mass (kg): mean (SD)	28.15 (4.84)
Total body water (kg): mean (SD)	41.24 (7.60)
Total blood volume (L): mean (SD)	5.04 (0.68)

APOE, apolipoprotein; Aβ, amyloid-β.

**Table 2:** Associations between Aβ and obesity indices

	Aβ-40				Aβ-42				Aβ ratio (40/42)			
	*B*	*P*	*P*_corr(FDR)_	Partial *η*^2^	*B*	*P*	*P*_corr(FDR)_	Partial *η*^2^	*B*	*P*	*P*_corr(FDR)_	Partial *η*^2^
BMI	−0.109	0.023	0.099	0.013	−0.142	0.003	0.018	0.022	0.057	0.211	0.305	0.004
WHtR	−0.106	0.029	0.105	0.012	−0.147	0.002	0.018	0.023	0.064	0.164	0.251	0.005

BMI and WHtR were separately analysed and adjusted for age, sex, batch and apolipoprotein genotype. Beta coefficients are standardized. Aβ, amyloid-β; FDR, false discovery rate; BMI, body mass index; WHtR, waist-to-height ratio; APOE, apolipoprotein E.

**Table 3:** Associations between Aβ and body composition

	Aβ-40				Aβ-42				Aβ ratio (40/42)			
	*β* (95% CI)	*P*	*P*_corr(FDR)_	Partial *η*^2^	*β* (95% CI)	*P*	*P*_corr(FDR)_	Partial *η*^2^	*β* (95% CI)	*P*	*P*_corr(FDR)_	Partial *η*^2^
BFM	−0.777 (−1.488 to −0.065)	0.032	0.105	0.011	−0.091 (−0.147 to −0.035)	0.001	0.018	0.025	0.014 (−0.002 to 0.03)	0.085	0.191	0.007
BCM	1.375 (−2.467 to 0.737)	0.289	0.376	0.003	−0.071 (−0.197 to 0.054)	0.265	0.363	0.003	0.004 (−0.031 to 0.04)	0.818	0.818	0.0001
ECM	−0.865 (−0.298 to 3.048)	0.107	0.214	0.007	0.131 (−0.001 to 0.261)	0.051	0.147	0.01	−0.01 (−0.05 to 0.027)	0.575	0.598	0.0008

BFM, BCM and ECM were analysed simultaneously in one model and adjusted for age, sex, batch, smoking and apolipoprotein genotype. Aβ, amyloid-β; FDR, false discovery rate; BFM, body fat mass; BCM, body cell mas; ECM, extracellular mass.

## Discussion

This study aimed to assess the relationship between plasma Aβ concentrations, anthropometric indices of obesity and body composition. In line with our hypotheses, obesity, operationalized as elevated BMI and WHtR, was significantly associated with lower plasma Aβ-42 concentrations. However, associations with Aβ-40 levels did not reach significance after accounting for multiple testing. Additionally, the analyses of BIA data revealed a significant inverse association between body fat mass and plasma Aβ-42 but, again, not with Aβ-40 concentrations. In contrast to our hypotheses, plasma Aβ concentrations were not significantly related to body cell mass, extracellular mass, total body water and total blood volume. Thus, the current results suggest a specific association between body fat and plasma Aβ concentrations. Similar results have been reported in older individuals, where a higher BMI was related to a lower cortical Aβ burden assessed using PET.^16^ The present results complement previous findings regarding other proteins of neuronal origin assessed in serum or plasma, e.g. lower concentrations of serum neurofilament light associated with higher BMI and body fat mass.^8,17^ For the potential future application of blood-based tests to quantify proteins of neuronal origin as a screening for neurodegenerative disorders, it is increasingly important to analyse peripheral factors that can affect or confound test performance. In support of our findings, it has also been observed that serum glial fibrillary acidic protein concentrations were lower in individuals with a higher BMI.^18^ A similar association has been found for phosphorylated tau at threonine 181 and 217 where a higher BMI was also related to lower concentrations.^19^ Apart from obesity and body composition, renal diseases were related to higher levels of phosphorylated tau.^20^ Similar effects have been observed for plasma Aβ-40, Aβ-42 and neurofilament light.^21^ It is important to consider such potential confounders as correction factors when applying ultrasensitive assays in the context of neurodegenerative diseases, e.g. in case of Alzheimer’s disease when the BMI of affected individuals often declines in the prodromal stage before they receive the diagnosis^9^ or when a renal disease is a comorbidity. It is furthermore necessary to account for the ApoE genotype since it affects plasma Aβ concentrations^22^ and, due to its role in fat metabolism, is also independently related to BMI^23^ as well as the accumulation of visceral adipose tissue.^24^

BMI alone is limited regarding the assessment of associations with Aβ levels since it does not discriminate between body fat and other body constituents such as muscle mass.^25^ However, the evaluation of body composition using BIA overcomes this limitation and allows the indirect classification of body fat, cell mass and extracellular mass. Although there exist more direct methods to assess body composition, i.e. computer tomography or hydrostatic weighting, estimates derived using BIA are in good agreement.^26^ A further limitation is that participants did not fast before plasma samples were collected, and parameters of renal dysfunctions were not available. Renal dysfunction^21^ as well as fasting can affect plasma parameters^27^ and a recent study^28^ on biomarkers related to Alzheimer’s disease concluded that further investigations are required to validate whether biomarker assessment in a fasting state could improve the accuracy in a diagnostic context. A major strength of the current study is the relatively large sample size and the random sampling of participants from the general population via the population register, and, in a diagnostic context, it has been shown that immunoassay-based methods are approaching the accuracy of immunoprecipitation-coupled mass spectrometry.^29^

## Conclusion

In conclusion, the results of the current study showed inverse associations between plasma Aβ-42 concentrations and obesity indices. Subsequent analyses revealed that body fat mass but no other body constituents were related to plasma Aβ concentrations. It is suggested to account for BMI as an easy-to-measure correction factor in diagnostic models evaluating the predictive value of blood-based Aβ.

## Data Availability

The data used in the manuscript are available upon reasonable request.
